# On Plugging the Teeth

**Published:** 1843-06

**Authors:** I. I. Greenwood


### ARTICLE III.

*On Plugging the Teeth.* By I. I. GREENWOOD, M. D., D. D. S.

I AM decidedly in favour of the operation of plugging the teeth, in those cases where it can be performed with ease to the patient and a fair prospect that it will be a benefit to the organs, but should feel myself in duty bound to discourage it when the operation must necessarily be attended with much pain and affords little hope of saving the teeth. In such cases, even should the patient be desirous of having the operation performed, I am of opinion that it would be the height of folly to acquiesce, as the surgeon-dentist always loses more in practice and reputation by the performance of one useless, painful operation, than he will recover in many useful and successful ones. Within the last ten years, the science of dentistry has so far advanced that no practitioner can plead any excuse, when about to perform an operation that may be so useful to a decaying tooth, for not having previously ascertained the exact state and condition of the organ, and the probability, amounting to almost certainty, that it can be done with entire satisfaction to himself and lasting benefit to his patient. I am well aware that teeth may, sometimes, be filled to advantage even when considerable pain must be the consequence to the patient; yet, at the same time, I would caution all good practitioners not to entertain too sanguine hopes that pain so caused will be of only temporary duration, as I have found, by experience, that abscesses of the medullary canal and diseased alveoli have often resulted from filling of the teeth, attended and followed by considerable pain. When the means of cure are so well known and so certain, in the present state of the science, there can be no excuse for not foreseeing and guarding against any such disastrous results. Dr. Spooner, and other eminent surgeon-dentists, have given sure and plain remedies in the application of arsenic to the diseased medulla, and my own experience, for years, in the successful use of this mineral to destroy the nerve of a tooth, fully confirms the opinion of others in regard to its high value.

During my practice of some twenty-seven years, I have used, in plugging defective teeth, the six following minerals: lead, tea lead, tin, silver, platina, and pure gold.

*Lead.*—Where no other metal could be used, I have adopted lead, on account of its softness, and its quality of speedy compaction—particularly when the nerve of the tooth was very sensitive, and it would not answer to press too severely upon the organ—using it only for the tender teeth, as this, with all other metals except gold, must be excluded from operations on the incisors, the cuspidati and the bicuspides. Lead may be used in the form of leaf or wire, and should be kept in a square, dry box, about the size of a leaf of foil, (as all other minerals used in plugging should be,) so as to keep it clean, dry and ready for use at a moment's warning, being, in this position, more accessible and easier to handle than in any other way.

*Tea Lead.*—This material comes from China, affixed to the papers which envelope the teas. By rubbing it in warm water, it becomes detached from the paper, and thus can be used as any other foil. In this form, as a medium to fill the teeth, I regard it as being next to silver. It is softer than any other metal excepting lead, far preferable to common lead, and in many cases holds well, and, as I have often known it, lasts for years.

*Tin Foil.*—This is a metal used by the looking-glass manufacturers, in silvering the backs of their mirrors, and, as there is a great variety of quality, I would advise the dentist to select the thinnest and the softest, as well as that which most resembles silver in brightness of colour. It is very much used by the faculty, answering very well to plug large cavities, when the patient is not willing to incur much expense. It answers well in preventing pain in the organ, and excludes the particles of food retained in mastication. It should only be used in the molar teeth, on account of its liability to oxidate, and to discharge a bluish matter which will stain the organs of the same hue. It is also liable, in the lapse of time, from the continued action of the septic acid, where the constitution of the patient inclines to an excess of that fluid in the mouth, to become absorbed; but, when the constitution of the subject is good, and the mouth dry, it will last for many years.

*Silver Foil,* when pure, is much better than tin foil, especially when well annealed so as to be soft and pliable; it will not oxidate as soon as tin, but will discharge a colouring matter, and give the teeth a bluish tint. It answers for operations on the molares; but I would not recommend its use upon any of the other teeth.

*Platina,* or white gold, as it is sometimes called, I should recommend in plugging the molar teeth, if it could be procured sufficiently pure and malleable as a foil, and if it could be used with the same ease as gold; yet, whenever I have made use of this mineral, either as filling for cavities in teeth or as pivots for artificial teeth, I have ever found it to turn all the substances with which it came in contact of a bluish colour; and, besides, it does not possess the ductility of gold, which is a most serious objection against its use. Could it be procured sufficiently pure, and as soft and pliable as gold, and divested of its liability to throw out colouring matter when acted upon by the juices of the mouth, I should pronounce it the king of metals, as a material for filling the teeth; but, for that purpose, *pure gold* is undoubtedly the sovereign of minerals.

*Pure Gold* is prepared by the gold beaters, from gold of from twenty-two to twenty-four carats fineness—the finer and more ductile the better—and it is, when well prepared, as easy to compress as lead, adheres much better, and has this eminent advantage over other metals, that it resists oxidation, and imparts no colour to the enamel of the tooth filled with its substance. In plugging the incisores, I prefer this metal on account of its greater durabitity. I have seen teeth, plugged by my father, in good preservation after forty years' use, and have no doubt when the operation is well performed, that the filling will last firm in the cavity as long as the tooth itself remains in its socket. I cannot speak too highly of this mineral, and would recommend to all those who may have decayed teeth to fill, and require the assistance of a dental surgeon, to insist upon the use of pure gold in every such operation, as being in the end the most economical, as well as lasting. I am much gratified that many of the most eminent among the faculty of dental surgery have totally excluded from their operations in filling teeth every metal except pure gold foil.

